# Vulgarin, a Sesquiterpene Lactone from *Artemisia judaica*, Improves the Antidiabetic Effectiveness of Glibenclamide in Streptozotocin-Induced Diabetic Rats via Modulation of PEPCK and G6Pase Genes Expression

**DOI:** 10.3390/ijms232415856

**Published:** 2022-12-13

**Authors:** Hassan N. Althurwi, Gamal A. Soliman, Rehab F. Abdel-Rahman, Reham M. Abd-Elsalam, Hanan A. Ogaly, Mohammed H. Alqarni, Faisal F. Albaqami, Maged S. Abdel-Kader

**Affiliations:** 1 Department of Pharmacology, College of Pharmacy, Prince Sattam Bin Abdulaziz University, Al-Kharj 11942, Saudi Arabia; 2 Department of Pharmacology, College of Veterinary Medicine, Cairo University, Giza 12613, Egypt; 3 Department of Pharmacology, National Research Centre, Giza 12622, Egypt; 4 Department of Pathology, College of Veterinary Medicine, Cairo University, Giza 12613, Egypt; 5 Department of Ecosystem and Public Health, Faculty of Veterinary Medicine, University of Calgary, Calgary, AB T3R 1J3, Canada; 6 Department of Chemistry, College of Science, King Khalid University, Abha 61421, Saudi Arabia; 7 Department of Biochemistry, College of Veterinary Medicine, Cairo University, Giza 12613, Egypt; 8 Department of Pharmacognosy, College of Pharmacy, Prince Sattam Bin Abdulaziz University, Al-Kharj 11942, Saudi Arabia; 9 Department of Pharmacognosy, College of Pharmacy, Alexandria University, Alexandria 21215, Egypt

**Keywords:** vulgarin, streptozotocin, glibenclamide, PEPCK, G6Pas, rat

## Abstract

The current investigation assessed the effect of the eudesmanolid, Vulgarin (VGN), obtained from *Artemisia judaica* (*A. judaica*), on the antidiabetic potential of glibenclamide (GLB) using streptozotocin (STZ) to induce diabetes. Seven groups of rats were used in the study; the first group received the vehicle and served as normal control. The diabetic rats of the second to the fifth groups were treated with the vehicle (negative control), GLB at 5 mg/kg (positive control), VGN at 10 mg/kg (VGN-10) and VGN at 20 mg/kg (VGN-20), respectively. The diabetic rats of the sixth and seventh groups were administered combinations of GLB plus VGN-10 and GLB plus VGN-20, respectively. The diabetic rats treated with GLB plus VGN-20 combination showed marked improvement in the fasting blood glucose (FBG), insulin and glycated hemoglobin (HbA1c), as well as the lipid profile, compared with those treated with GLB alone. Further, the pancreatic tissues of the diabetic rats that received the GLB+VGN-20 combination showed superior improvements in lipid peroxidation and antioxidant parameters than those of GLB monotherapy. The insulin content of the β-cells was restored in all treatments, while the levels of glucagon and somatostatin of the α- and δ-endocrine cells were reduced in the pancreatic islets. In addition, the concurrent administration of GLB+VGN-20 was the most effective in restoring PEPCK and G6Pase mRNA expression in the liver. In conclusion, the results demonstrated that the GLB+VGN-20 combination led to greater glycemic improvement in diabetic rats compared with GLB monotherapy through its antioxidant effect and capability to modulate PEPCK and G6Pase gene expression in their livers.

## 1. Introduction

The World Health Organization (WHO) expects that up to 366 million individuals will suffer from diabetes mellitus (DM) by 2030 [1]. Several members of the genus *Artemisia* are used in folk medicine for the management of the impaired blood glucose levels of diabetic individuals [2]. Genus *Artemisia* is the source of a bioactive compound such as artemisinin, used as an antimalarial agent and also expresses profound cytotoxicity against cancer cell lines [3]. In the Arabian Gulf folk medicine, *A. judaica* is used for the management of many disorders, such as diabetes and parasitic infestation [4,5]. In Jordan, the infusion of *A. judaica* is used by the Bedouins for the management of diabetes and sexual weakness [6]. The use of *A. judaica* extract with glibenclamide resulted in greater glycemic improvement than GLB monotherapy in male Wistar rats [7]. A detailed study traced the hypoglycemic effect of *A. judaica* to the eudesmanolid, vulgarin (VGN) (judaicin), and its isomer epivulgarin [8].

In the current study, we evaluated the beneficial effects of using VGN and GLB combination compared with GLB monotherapy. The study comprises detailed biochemical, histopathological, immunohistochemical and gene expression evaluations.

## 2. Results

### 2.1. Effect on Fasting Blood Glucose (FBG) and Insulin Levels

**Figure 1:**
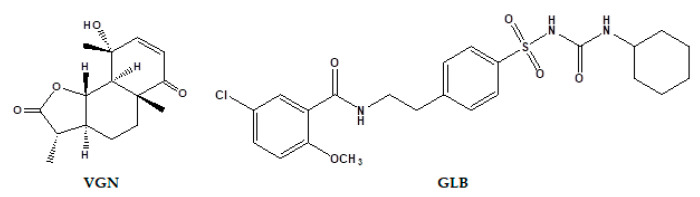
Structures of vulgarin (VGN) and glibenclamide (GLB).

The effect of GLB, VGN (Figure 1) and their combinations on FBG levels of STZ- diabetic rats are presented in Table 1. An increase in the FBG level persisted in diabetic rats throughout the whole experimental period (8 weeks). GLB treatment significantly reduced FBG levels in the diabetic rats throughout the experiment compared with the FBG levels of the diabetic control (DC) group; however, the parameter was not normalized (Table 1). The antidiabetic effect of the reference drug GLB was comparable to that of VGN-10, VGN-20 and GLB+VGN-10. Notably, rats treated with the GLB+VGN-20 combination caused a more significant decrease in the FBG level compared to GLB monotherapy. This combination normalizes FBG level (94.7 ± 5.56 mg/dL) after 8 weeks of treatment compared with 97.8 ± 4.96 mg/dL in the normal control (NC) group.

Table 2 summarizes the serum insulin levels at the end of the second, fourth and eighth week of the treatment periods. In comparison with the NC group, the serum insulin levels in the DC rats showed significant reductions after 2, 4 and 8 weeks of the experimental period. These significantly reduced levels were successfully reversed in the diabetic rats subjected to treatment with GLB monotherapy. The level of insulin in diabetic rats treated with VGN-10, VGN-20 and GLB+VGN-10 was significantly elevated in comparison with GLB monotherapy; however, the insulin level was still much lower than the NC group (Table 2). Importantly, the most significant elevation of insulin levels in diabetic rats was observed in the group treated with GLB+VGN-20 for 8 weeks. The blood insulin level of diabetic rats exposed to this combination is comparable to those of NC rats.

### 2.2. Effect on Total Hemoglobin (Hb) and Glycosylated Hemoglobin (HbA1c) Levels

The blood level of Hb and HbA1c in normal and diabetic rats are presented in Table 3. DC rats showed a significant reduction in Hb (9.9 ± 0.25 g/dL) and an increase in HbA1c (9.0 ± 0.32%) levels when compared with normal levels (14.6 ± 0.71 g/dL and 4.9 ± 0.34%, respectively). Over the 8-week treatment period, HbA1c percentages improved from 9.0 ± 0.32% in DC rats to 6.7 ± 0.30% with GLB monotherapy. HbA1c% in the diabetic GLB-treated rats did not differ much from those of the VGN-10, VGN-20 and GLB+VGN-10 treated animals. Interestingly, the GLB+VGN-20 combination reduced the elevated levels of blood HbA1c observed in DC and GLB monotherapy groups. GLB+VGN-20 combination almost normalized the levels of both Hb and HbA1c. 

### 2.3. Effect on Blood Lipid Profile

Serum lipid profiles in the control and treated groups are presented in Table 4. Serum triglycerides (TG), total cholesterol (TC) and low-density lipoprotein (LDL) levels were significantly elevated, and high-density lipoprotein (HDL) level was decreased in DC rats when compared with the NC group. Administration of GLB, VGN-10, VGN-20 and the GLB+VGN-10 combination significantly reduced the levels of TG, TC and LDL-C in diabetic rats compared with the DC group. However, their levels remained significantly elevated when compared with NC animals. Furthermore, HDL-C levels were significantly increased when compared with the DC group. Remarkably, treatment with GLB+VGN-20 combination tends to normalize and improve serum lipid profile in diabetic rats. 

### 2.4. Effect on Oxidative Stress and Lipid Peroxidation (LPO) Markers in the Pancreatic Tissues

Table 5 demonstrates the effect of GLB and VGN on oxidative stress and lipid peroxidation markers in the pancreatic homogenates of rats. STZ-induced diabetes significantly reduced the levels of pancreatic antioxidant enzymes such as SOD, GPx and CAT and increased the malondialdehyde (MDA) level in the DC group in comparison with the NC group. The diabetic rats exposed to GLB monotherapy showed significantly increased levels of SOD, GPx and CAT, whereas MDA levels were significantly decreased as compared with DC rats. The results indicated that the antioxidant activities of VGN-10, VGN-20 and GLB+VGN-10 are comparable to the GLB treated group. Interestingly, treatment with the GLB+VGN-20 combination showed enhancements in the pancreatic levels of SOD, GPx, CAT and MDA and almost normalized their levels. 

### 2.5. Histopathological Examination of Pancreas

**Figure 2:**
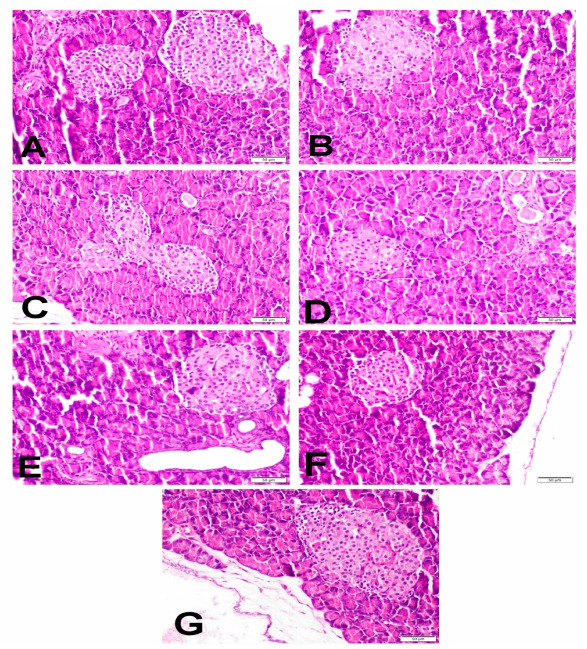
The histopathological photomicrographs of the pancreases in all experimental groups: (**A**) NC group showing normal islets composition; (**B**) STZ-control group showing marked reduction in the number, necrosis and apoptosis of β-cells and distortion of the islet’s composition; (**C**,**D**) GLB and VGN-10 showing mild reduction in the number of β-cells; (**E**–**G**) VGN-20, GLB+VGN-10 and GLB+VGN-20 showing marked improvement of the β-cells number with single cell necrosis.

The normal pancreatic architecture of the islets of Langerhans, the exocrine gland and the pancreatic duct was recorded in the NC group (Figure 2A). There were well-arranged islets of Langerhans with a central core of Beta (β) cells and a peripheral layer of both Alpha (α) and Delta (δ) cells. In the DC group, a marked decline in the number of centrally located β-cells with the incidence of apoptosis and necrosis in the islets of Langerhans was observed (Figure 2B). Moreover, a severe dilatation of the pancreatic duct with papillary hyperplastic epithelial cells was also observed. Nevertheless, GLB (Figure 2C), VGN-10 (Figure 2D) and VGN-20 (Figure 2E) groups showed a moderate reduction in the number of β-cells. However, GLB+VGN-10 (Figure 2F) and GLB+VGN-20 (Figure 2G) revealed a marked increase in the number of β-cells with the restoration of the islets of Langerhans’s normal architecture.

### 2.6. Immunohistochemistry of Insulin, Glucagon and Somatostatin Contents of Islets of Langerhans

**Figure 3:**
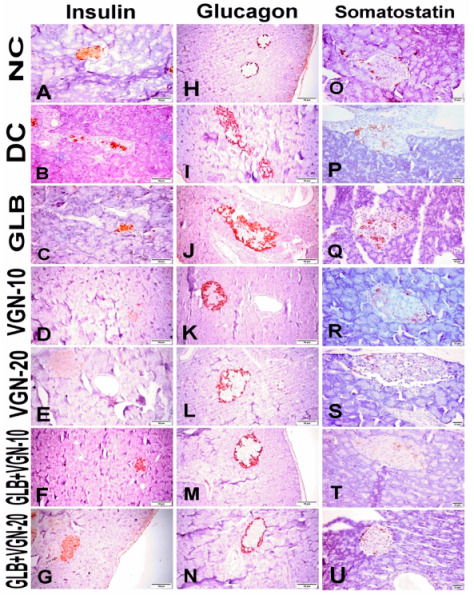
Representative anti-insulin, anti-glucagon and anti-somatostatin immunohistochemistry in the pancreases of all experimental groups: (**A**–**G**) anti-insulin protein expression; (**H**–**N**) anti-glucagon protein expression; (**O**–**U**) anti-somatostatin protein expression; (**A**,**H**,**O**) NC group; (**B**,**I**,**P**) DC group; (**C**,**J**,**Q**) GLB group; (**D**,**K**,**R**) VGN-10 group; (**E**,**L**,**S**) VGN-20 group; (**F**,**M**,**T**) GLB+VGN-10 group; (**G**,**N**,**U**) GLB+VGN-20 group.

**Figure 4:**
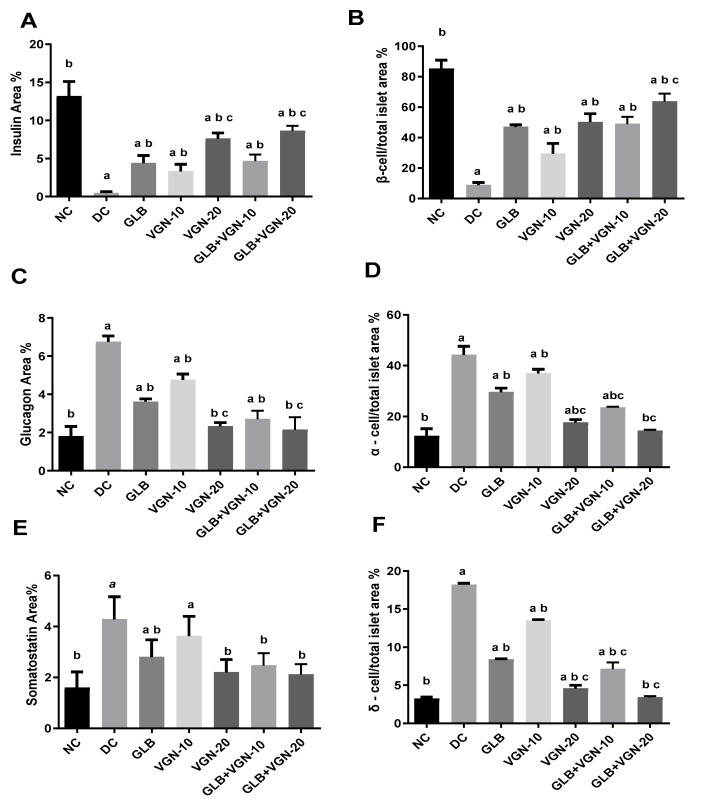
Morphometric analyses of islets of Langerhans’ composition: (**A**) insulin content of β-cells; (**B**) β-cell/total islet area %; (**C**) glucagon content of α-cells %; (**D**) α-cell/total islet area %; (**E**) glucagon content of δ-cells %; (**F**) δ-cell/total islet area %. Values are expressed as the mean ± S.E (n = 6). ^a,b,c^ refer to significant changes at *p* ≤ 0.05 with respect to NC, DC and GLB groups, respectively.

Immunohistochemical analysis of insulin, glucagon and somatostatin protein expressions revealed strong central diffuse insulin immune-positive staining in β-cells, peripheral glucagon immune-positive staining in α-cells and peripheral somatostatin immune-positive staining in δ-cells that formed an incomplete circle in the islets of Langerhans in the NC group (Figure 3A,H,O). Immunohistochemical analyses of the different protein expressions, as well as the morphometric analysis of the islets of Langerhans, were summarized in Figure 4. A significant decline in the insulin content of β-cells and β-cell/total islet area % was recorded in the DC group (Figure 3B and Figure 4A,B) when compared with the NC group. In addition to that, a significant increase in both glucagon and somatostatin contents in the islets of Langerhans, as well as α-cell/total islet area % and δ-cell/total islet area %, were also recorded in the DC group compared with the NC group (Figure 3I,P and Figure 4C–F). On the contrary, a significant increase in the insulin contents of the β-cells, β-cell/total islet area % with a significant decrease in glucagon, somatostatin contents, α-cell/total islet area % and δ-cell/total islet area % was recorded in GLB (Figure 3C,J,Q), VGN-10 (Figure 3D,K,R), VGN-20 (Figure 3E,L,S), GLB+VGN-10 (Figure 3F,M,T) and GLB+VGN-20 (Figure 3G,N,U) treated group when compared with STZ group. VGN-20 combination with GLB exhibited the best improvement in islet morphology and insulin content of the β-cells among the other groups. 

### 2.7. Quantitative Real-Time PCR (qRT-PCR) 

**Figure 5:**
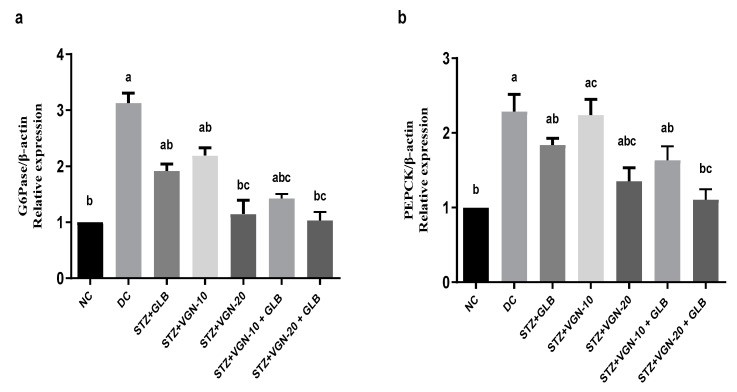
VGN downregulates STZ-induced PEPCK and G6Pase expression in rat liver tissues: total RNA was extracted, and mRNA expression of G6Pase (**a**) and PEPCK (**b**) was analyzed by quantitative RT-PCR. Data are expressed as fold change over basal relative to β-actin reference gene (mean ± SEM) (n = 6). ^a,b,c^ refer to significant changes at *p* ≤ 0.05 with respect to NC, DC and GLB groups, respectively.

The effect of VGN on the expression of the hepatic gluconeogenesis-related genes was assessed using qRT-PCR. As shown in Figure 4, the DC group showed a significant increase in PEPCK and G6Pase mRNA levels as compared with normal control rats. PEPCK and G6Pase (*p* < 0.05) mRNA expression was significantly lower in groups subjected to VGN VGN-20 monotherapy and combination therapy (GLB+VGN-10, GLB+VGN-20) compared with the DC group. The modulatory effect of VGN on gluconeogenic gene expression was comparable to the reference drug, GLB. Remarkably, GLB+VGN-20 treatment was superior in the reduction of G6Pase and PEPCK mRNA levels, restoring the levels to that of the NC (Figure 5). These findings indicated that the effect of VGN on gluconeogenesis was associated with the regulation of the key gluconeogenic enzymes, PEPCK and G6Pase.

**Figure 6:**
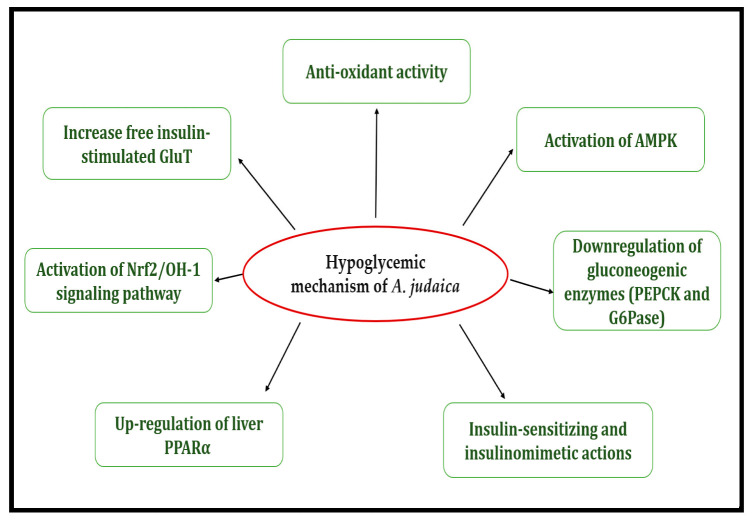
Different mechanisms of action of the antidiabetic effects of *Artemisia* plants.

In summary, Figure 6 shows several different mechanisms of action to describe the potential antidiabetic effect of *Artemisia* plants.

## 3. Discussion

The isolated compound was identified as VGN (barraelin, judaicin, tauremisin C) based on the spectroscopic data, including 1D, 2D-NMR and ESIHRMS (Figures S1–S5), and compared with the literature [9].

STZ is one of the most commonly used compounds in animal models to induce diabetes. Upon STZ treatment, the pancreatic β-cells undergo death as a result of DNA alkylation and fragmentation, induced by ROS production, leading to reduced synthesis and release of insulin [10]. The action of STZ is selective to pancreatic cells that secrete insulin. As a result, there are fewer active pancreatic cells, and DM develops [11].

### 3.1. Effect on Fasting Blood Glucose (FBG) and Insulin Levels

The glycemic state of diabetic animals was assessed by the levels of serum glucose and insulin. Table 1 and Table 2 demonstrate the effects of eight weeks’ treatment with GLB alone or in combination with either VGN-10 or VGN-20 on serum levels of FBG and insulin in diabetic rats, respectively. As expected, high FBG and low insulin levels were observed in the DC rats when compared with NC rats. On the other hand, GLB treatment significantly reduced FBG levels by 59.88% and increased insulin levels by 70.08% when compared to basal values in the NC. These results support the notion of GLB’s ability to stimulate insulin release from β-cells and improve type 2 diabetes [12]. The action of GLB is believed to be mediated by its ability to bind to the sulfonylurea receptor 1, a regulatory subunit of the ATP-sensitive potassium channels in the pancreatic β-cells [13]. Interestingly, the hypoglycemic potential of GLB is maximized when combined with VGN-20. This combination was found to be the most effective in the normalization of FBG and insulin levels throughout the experimental period. More remarkably, these beneficial effects of the GLB+VGN-20 combination occur without an increased risk of hypoglycemia, suggesting effective and proper glycemic control. 

### 3.2. Effect on Total Hemoglobin (Hb) and Glycosylated Hemoglobin (HbA1c) Levels

The level of HbA1C is increased in diabetic rats as a result of persistent hyperglycemia, which causes glycation of hemoglobin. Therefore, HbA1c level is used as an important marker to monitor glycemic control due to its strong correlation with blood glucose levels [14]. Our results reveal that GLB, VGN and their combinations confer protection against hemoglobin glycation. Diabetic rats treated with GLB, VGN-10, VGN-20 and GLB+VGN-10 showed a significant decrease in the percentage of HbA1c but did not essentially improve the rate of glycosylation compared with NC levels indicating incomplete glycemic control. HbA1c values of <7% reflect good glycemic control in most diabetic patients [15]. HbA1c percentage was well controlled in the GLB+VGN-20 treated rats when compared with animals treated with GLB alone. In addition, the HbA1c percentage returned to the normal range following 8 weeks of treatment with the GLB+VGN-20 combination (5.2 ± 0.27%), suggesting an effective control of hyperglycemia by the GLB+VGN-20 combination. This also suggests that this combination could confer long-term control of DM, which might delay or even prevent the development of complications in diabetic individuals.

### 3.3. Effect on Blood Lipid Profile

Hyperlipidemia and diabetes are associated as it is well documented that insufficient control of glucose in diabetic patients results in disturbance in the serum lipid profile [16,17]. Our study revealed a significant increase in the serum levels of TG, TC and LDL-C in STZ-induced diabetic rats compared to NC rats. HDL-C was significantly reduced in DC rats when compared to the NC group. On the other hand, treatment with GLB, VGN-10, VGN-20 and GLB+VGN-10 attenuated all the changes in lipid profile, and this observation might be related to their ability to reduce sugar level; however, these treatments failed to bring the lipid profile to the normal levels. Treatment with GLB+VGN-20 combination had a greater effect on regulating serum lipid profile than GLB monotherapy. This combination was able to restore the lipid profile back to those seen in NC rats reflecting the antidiabetic potential of this combination. This effect could be attributed to the ability of the combination therapy to increase insulin secretion from pancreatic β-cells. This increase in insulin secretion also induces fatty acid synthesis and incorporation into TG in the liver and adipose tissue. 

### 3.4. Effect on Oxidative Stress and Lipid Peroxidation (LPO) Markers in the Pancreatic Tissues

Previous reports indicated that oxidative stress is a hallmark of the damaging effects of STZ in pancreatic tissues leading to increase apoptosis in target cells [18]. Mounting evidence reveals a strong correlation between DM and oxidative stress. The oxidative stress results in either increased production of free radicals and/or reduction in antioxidant enzyme activities [19]. The antioxidant capacities of the cells are mediated, in part, by the action of several antioxidant enzymes, including SOD, GPx and CAT. These enzymes are considered the first line of defense against oxidative stress. In our study, SOD, GPx and CAT activities were significantly reduced in DC compared to the NC group. Treatment of diabetic rats by GLB increased the antioxidant enzyme activities in pancreatic tissues. In accordance with our findings, GLB proved to counteract ROS production and attenuated oxidative stress [20]. The antioxidant effects of VGN-10, VGN-20 and GLB+VGN-10 were comparable to that observed in GLB treated group. However, the antioxidant efficacy of the GLB+VGN-20 combination was superior to the beneficial effects of GLB monotherapy. In addition, GLB+VGN-20 combination therapy abolished the changes in antioxidant enzyme activities mediated by diabetes. This suggests a synergistic effect of GLB+VGN-20 against STZ-induced oxidative stress in diabetes.

The level of MDA, a biomarker of the lipid peroxidation process, in plasma and tissues was elevated in DC rats [21]. In agreement with this observation, our findings showed that MDA levels increased significantly in the pancreatic tissues of STZ-treated rats, suggesting severe lipid peroxidation when compared with NC rats. This could be, in part, due to elevated levels of ROS and oxidative stress in these tissues [22]. On the other hand, the pancreatic content of MDA in groups treated with GLB, VGN-10, VGN-20 and GLB+VGN-10 was significantly decreased when compared to the DC group. This reduction could be an indication of improving the enzymatic and non-enzymatic antioxidant defense mechanisms [23]. In addition, 8-week treatment with GLB+VGN-20 successfully normalized the disturbed levels of MDA in the pancreatic tissue of diabetic rats. Altogether, these results suggest a synergistic effect of GLB and VGN-20 on reducing pancreatic lipid peroxidation in STZ diabetic rats.

### 3.5. Histopathological Examination of Pancreas and Immunohistochemistry of Insulin, Glucagon and Somatostatin Contents of Islets of Langerhans

As previously described, many mechanisms explained β-cells toxicity induced by the administration of STZ, such as free radicals production and nitric oxide donation [24]. This means that the insulin content of the islet of Langerhans would decline with an increase in glucagon and somatostatin content of α-cells and δ-cell of these islets as observed in the DC group. In the present study, the different medicaments revealed improvement in the islets morphology and an increase in the insulin content of β-cells of the pancreatic islets, especially with GLB+VGN-20 treatment, which appeared in the form of reducing the hyperglycemia in this group. Therefore, the combination between GLB and VGN may protect β-cells from toxicity induced by STZ. 

### 3.6. Effect on the Gluconeogenic Enzymes PEPCK and G6Pase Gene Expression

Based on accumulating evidence, the rate of hepatic gluconeogenesis is elevated in diabetic conditions. Therefore, regulation of the gluconeogenic pathway could provide an efficient mechanism for controlling blood glucose levels [25,26]. In the current study, the expression of two rate-limiting enzymes in hepatic gluconeogenesis was studied. The enzyme PEPCK helps to catalyze the conversion of oxaloacetate to phosphor–pyruvate, the first step of gluconeogenesis [27]. G6Pase is another rate-limiting enzyme catalyzing the final step of gluconeogenesis by hydrolysis of glucose 6-phosphate into glucose [28]. Our findings revealed that the DC group showed an increased expression of PEPCK and G6Pase genes (Figure 5a,b). These findings come in line with those previously reported [26,29,30]. Under normal conditions, insulin strongly represses the expression of endogenous PEPCK and G6Pase transcription. On the other hand, upregulation of these gluconeogenic genes results in loss of insulin sensitivity in the tissue. Here, we report that VGN could mimic the effect of insulin on gluconeogenic enzymes. Treatment with VGN, GLB or their combination exerts significant downregulation of hepatic G6Pase and PEPCK expression (Figure 6). Thus, in addition to the different mechanisms of action of the antidiabetic effects of *Artemisia* plants (Figure 6), the recorded hypoglycemic effect of VGN could be partially attributed to the reduction of hepatic glucose output.

## 4. Materials and Methods

### 4.1. Plant Material 

The plants of *Artemisia judaica* L. were collected from the Huraymila region about 85 km West of Riyadh city in January 2018. The plant materials were identified by the taxonomist of the MAP-PRC at the College of Pharmacy at King Saud University, Riyadh, Saudi Arabia, Dr. Mohammad Atiqur Rahman. A voucher specimen (#16723) was kept at the herbarium of this center. 

### 4.2. Extraction and Isolation

The dried ground aerial parts (5 kg) of *A. judaica* were extracted by 95% ethanol (50 L) at room temperature until exhaustion. The solvent of the resulting combined extract was evaporated under a vacuum to give a viscous dark green residue with an aromatic odor. The total extract was dispersed in 1.5 L of 40% aqueous ethanol and subjected to liquid–liquid fractionated using petroleum ether (800 mL × 3), chloroform (800 mL × 4) and ethyl acetate (800 mL × 3). The left aqueous later was designated as an aqueous fraction. 

#### Chloroform Fraction

Part of the chloroform fraction (40 g) was chromatographed on a Sephadex LH20 column (150 × 10 cm i.d., 1000 g), eluting with chloroform/acetone 75:25 followed by a gradual increase in acetone contents by 25% until reaching 100% acetone, then a gradient of acetone/methanol was used until 100% methanol was reached by increasing methanol by 25%. Fractions of 150 mL were collected, and similar fractions were combined to give 6 major fractions (A–F) based on their TLC profile. Fraction C (10.79 g) eluted with chloroform/acetone 25:75 was purified using a silica gel column (90 × 5 cm i.d., 400 g). Elution started with chloroform and then chloroform/methanol mixtures in a gradient elution system. Fractions 10–25 (5.2 g) provided 3.89 g of vulgarin after repeated crystallization from methanol. Fractions 27–31 (2.3 g) provided 900 mg of *epi*-vulgarin after repeated crystallization from methanol.

### 4.3. Animals

Male Wistar rats (180–200 g) procured from the Animal House of the National Research Centre (NRC), Cairo, Egypt, were maintained in controlled conditions. Seven days prior to the study, the animals were acclimated to the laboratory environments. Rats were kept in controlled conditions at a temperature of 26 ± 2 °C, 12 h day/night cycles, and were offered food and water ad libitum. Experimental procedures of the study were approved by the Institutional Animal Care and Use Committee, Cairo University (approval number: CU-II-F-86-18), complying with the recommendations of the National Institutes of Health Guide for Care and Use of Laboratory Animals (Publication No. 85-23, revised 1985).

### 4.4. Induction of Experimental Diabetes

Experimental diabetes was produced by a single dose of 45 mg/kg body weight of STZ (Merck& Co, Rahway, NJ, USA) in 0.01 M citrate buffer (pH 4.5) via intraperitoneal injection [7]. Diabetes was checked in blood samples collected from the tail veins of rats by measuring FBG levels using a blood glucose meter after three days (Accu- Check Performa, Roche Diagnostic, Germany). Rats were considered diabetic and selected for the study when their blood glucose level exceeded 250 mg/dL [31].

### 4.5. Experimental Design

Seven groups of weight-matched rats (six animals each) were divided as follows: Negative Control (NC); diabetic control (DC) induced by a single dose of 45 mg/kg body weight of STZ; diabetic rats treated with glibenclamide at 5 mg/kg (GLB); diabetic rats treated with Vulgarin at 10 mg/kg (VGN-10); diabetic rats treated with VGN at 20 mg/kg (VGN-20); diabetic rats treated with GLB and VGN at 10 mg/kg (GLB+VGN-10); and diabetic rats treated with GLB and VGN at 20 mg/kg (GLB+VGN-20). The standard (GLB) and the tested compound were administered once daily through oral gavage for 8 weeks. Weekly body weight variations were monitored for all the experimental animals.

### 4.6. Effect on Fasting Blood Glucose (FBG) and Insulin Levels

Blood samples were withdrawn from the retro-orbital venous plexus of overnight fasted animals under pentobarbital sodium (35 mg/kg, ip) anesthesia into tubes containing sodium fluoride at different time points (0, 2, 4 and 8 weeks of treatment). Serum was obtained by centrifugation of blood samples for 20 min at 5000 rpm. Serum’s FBG and insulin levels were estimated using commercially available Spinreact ELISA kits (Spinreact, Girona, Spain) and Cobas ELISA kits (Roche, Brussel, Belgium) following the manufacturer’s manual, respectively.

The hypoglycemic activity of GLB, VGN and their combination was calculated as a percentage of glucose reduction according to the following formula [32]:

% of Blood glucose reduction at time t = [(a − b)/a] × 100

where a is the initial blood glucose level, and b is the blood glucose level at time t.

### 4.7. Effect on Total Hemoglobin (Hb) and Glycosylated Hemoglobin (HbA1c) Levels

Another blood sample was similarly obtained from each overnight fasted rat at the end of the experiment (after 8 weeks of the medication period) into heparinized tubes for estimation of total Hb [33] and HbA1c [34].

### 4.8. Effect on Blood Lipid Profile

The lipid profile of animals (triglycerides (TGs), total cholesterol (TC), high-density lipoprotein cholesterol (HDL-C) and low-density lipoprotein (LDL-C) was assessed using blood samples obtained at the end of the 8th week of treatments as previously reported [35,36,37,38].

### 4.9. Tissue Collection

At the end of the experimental period, animals were euthanized via cervical decapitation, and pancreatic tissues were harvested and split into two halves.

### 4.10. Effect on Oxidative Stress and Lipid Peroxidation (LPO) Markers in the Pancreatic Tissues

One-half of the pancreas tissue was washed with ice-cold saline immediately and weighed. Then, it was kept at −80 °C until further analysis. After homogenizing pancreatic tissues, using a homogenizer, “Medical equipment, MPW-120, Poland”, in 0.1 M Tris-HCl (pH 7.4) buffer, homogenates were centrifuged at 1700 rpm for 10 min. The supernatants were separated, collected and maintained at −20°C for subsequent biochemical analysis. The supernatants of the pancreas homogenates were quantified for SOD, GPx and CAT spectrophotometrically according to the commercial instructions of the kits. Lipid peroxidation was determined by measuring MDA content in the pancreatic tissues according to the procedure of Jain et al. [39].

### 4.11. Histopathological Examination of Pancreas

Specimens from pancreases of all experimental groups were collected and fixed in 10% neutral buffered formalin for 24–48 h. The fixed tissue specimens were further processed to obtain 4 µm paraffin embedding tissue sections, then routinely stained with Hematoxylin and Eosin (H&E) stain [26].

### 4.12. Immunohistochemistry of Insulin, Glucagon and Somatostatin Contents of Islets of Langerhans

The test was performed according to Alsharif et al. [40] and Althurwi et al. [41]. Deparaffinization, rehydration and antigenic retrieval with sodium citrate were performed on all tissue sections following the methods of Abu-Elala et al. [42]. Tissue sections were washed three times with tris buffer saline (TBS) followed by incubation with one of the following primary antibodies; mouse monoclonal insulin (Sc-8033; Santa Claus, IN, USA) at 1:100 dilutions, mouse monoclonal anti-glucagon (MABN238; Cobas ELISA kits, Roche, Brussel, Belgium) at 1:500 dilution and rat monoclonal anti-somatostatin (MAB354; Millipore, Merck, Darmstadt, Germany) at 1:100 for overnight at the humid chamber. All tissue sections were washed two times with TBS and then incubated with a secondary sheep anti-mouse antibody (AQ300D; Millipore, Merck, Darmstadt, Germany) or goat anti-rat antibody (AP136P; Millipore, Merck, Darmstadt, Germany) according to the kind of primary antibody for 15 min followed by two times washing and streptavidin peroxidase incubation (Thermo Scientific, Waltham, MA, USA). Finally, all tissue sections were stained with 3,3′-diaminobenzidine tetrahydrochloride (DAB; Sigma, St. Louis, MO, USA) to visualize the reaction for 5–7 min, then counterstained with Mayer’s Hematoxylin. The islet morphometric analysis was performed by image J analysis software (Image J, version 1.46a, NIH, Bethesda, MD, USA). In each group, 7 microscopic fields were analyzed, and the percentage of the positive stained area (%) was calculated. In addition, the percent of insulin-positive β-cells/total islets area, α-cell/total islet area % and δ-cell/total islet area % were also calculated [43]. 

### 4.13. Quantitative Real-Time PCR (qRT-PCR) 

Separation and extraction of total RNA from frozen liver tissues were performed using an RNA extraction reagent (TRIzol^®^, Qiagen), and the RNA concentration and purity were estimated spectrophotometrically [44]. For cDNA synthesis, 1 μg RNA from each sample was used in 10 µL reverse transcription reaction with Oligo dT Primer (0.5 µL), RT Enzyme (0.5 µL), 5X reaction buffer (2 µL) and up to 10 µL RNase-Free dH2O (4.5 µL) according to the operating instructions of SuperScript IV VILO reverse transcriptase kit (Invitrogen). RT reaction condition was adjusted at 37 °C for 10 min, followed by 85 °C for 5 s. For quantitative PCR amplification of glucose 6-phosphatase (G6Pase) and phosphoenolpyruvate carboxykinase (PEPCK), a 25 µL reaction consisting of 2 µL cDNA, 10 µL Luminaries Color HiGreen SYBR master mix, 0.5 µL of each forward and reverse primer, 0.5 µL 50x ROX Reference Dye and up to 25 µL RNase-Free dH2O, according to the protocol recommended by the manufacturer (Thermo Scientific). The thermocycling condition was as follows: pre-denaturation (95 °C, 10 min), 40 cycling repeats of denaturation (95 °C, 10 s), annealing (56 °C, 15 s) and extension (72 °C, 20 s). The PCR primers sequences were as follows: G6Pase forward, 5’-GGATCTACCTTGCGGCTCACT-3’ and reverse, 5’-TGTAGATGCCCCGGATGTG-3’; PEPCK forward, 5’-GTGTCATCCGCAAGCTGAAGA-3’ and reverse, 5’-CTTTCGATCCTGGCCACATCT-3’; and β-actin forward, 5’-ATGGTGGGTATGGGTCAG -3’ and reverse, 5’-CAATGCCGTGTTCAATGG-3’ [45]. Each assay was carried out in triplicate. Data analysis for calculation of the relative gene expression was performed using the 2-ΔΔCT method, where mRNA expression levels of target genes were normalized to the reference gene β-actin and expressed as a fold change in the NC level [46]. 

### 4.14. Statistical Analysis

Results were expressed as means ± SE. Comparisons between different groups were evaluated using one-way ANOVA followed by Dunnett’s multiple comparison tests (SPSS Program; Version 11.5, Tableau, Seattle, WA, USA). The difference was considered significant when *p* ≤ 0.05.

## 5. Conclusions

The present data led to the conclusion that GLB and VGN have a synergistic effect as the antidiabetic potential of their combination is stronger compared to GLB alone. The antidiabetic effect of the combination is most likely mediated via the antioxidant effect, the ability to restore the β-cells insulin content, as well as the reduction of both glucagon and somatostatin contents in the α- and δ-endocrine cells, respectively, in the pancreatic islets. Further, GLB and VGN could cause their synergistic antidiabetic interaction through modulation of PEPCK and G6Pase gene expression in the liver of diabetic rats.

The synergistic antidiabetic effect was superior and most significant in diabetic rats treated with the GLB+VGN-20 combination. Hence, GLB doses may require special attention when it is concomitantly administered with *A. judaica* to provide better control of the disease in the patients and to avoid any unexpected serious acute hypoglycemia.

## Figures and Tables

**Table 1:** Effect of GLB, VGN and their combinations on the FBG levels in STZ-induced rats.

Groups	FBG (mg/dL)						
	Baseline	2 Weeks		4 Weeks		8 Weeks	
	M ± SEM	M ± SEM	%	M ± SEM	%	M ± SEM	%
NC	95.7 ± 4.92	98.5 ± 4.39	−3.14	97.80 ± 4.25	−2.47	97.8 ± 4.96	−2.28
DC	344.2 ± 9.56 ^a^	361.5 ± 9.77 ^a^	−5.08	362.7 ± 12.57 ^a^	−5.31	314.0 ± 4.59 ^a^	8.40
GLB	344.3 ± 16.24 ^a^	189.0 ± 7.65 ^ab^	44.96	179.2 ± 10.77 ^ab^	48.12	138.3 ± 7.30 ^ab^	59.88
VGN-10	340.3 ± 11.30 ^a^	194.5 ± 11.04 ^ab^	43.04	184.5 ± 4.36 ^ab^	45.68	146.8 ± 3.27 ^ab^	56.77
VGN-20	345.5 ± 11.03 ^a^	170.8 ± 5.84 ^ab^	50.51	155.3 ± 6.24 ^ab^	55.10	117.5 ± 7.14 ^ab^	66.03
GLB+VGN-10	361.7 ± 15.99 ^a^	189.2 ± 9.99 ^ab^	47.70	167.5 ± 6.01 ^ab^	53.60	128.7 ± 4.42 ^ab^	64.33
GLB+VGN-20	349.8 ± 15.68 ^a^	160.7 ± 7.94 ^abc^	54.00	118.5 ± 4.96 ^abc^	66.08	94.7 ± 5.56 ^abc^	73.00

Values are expressed as Mean ± SEM of six animals in each group. ^a,b,c^ refer to significant changes at the corresponding time interval (*p* ≤ 0.05) with respect to NC, DC and GLB groups, respectively.

**Table 2:** Effect of GLB, VGN and their combinations on insulin levels in blood of STZ-induced rats.

Groups	Insulin (mIU/L)						
	Baseline	2 Weeks		4 Weeks		8 Weeks	
	M ± SEM	M ± SEM	%	M ± SEM	%	M ± SEM	%
NC	8.5 ± 0.47	8.6 ± 0.31	−1.56	8.5 ± 0.35	−0.22	8.6 ± 0.65	−1.68
DC	3.4 ± 0.22 ^a^	3.2 ± 0.15 ^a^	4.85	3.3 ± 0.16 ^a^	4.61	3.1 ± 0.14 ^a^	4.63
GLB	3.5 ± 0.13 ^a^	3.9 ± 0.27 ^ab^	−11.13	4.6 ± 0.26 ^ab^	−33.38	5.1 ± 0.28 ^ab^	−46.64
VGN-10	3.3 ± 0.15 ^a^	3.6 ± 0.09 ^ab^	−10.94	4.2 ± 0.19 ^ab^	−27.95	4.6 ± 0.22 ^ab^	−41.64
VGN-20	3.4 ± 0.16 ^a^	4.3 ± 0.27 ^ab^	−28.32	5.3 ± 0.25 ^ab^	−56.78	5.9 ± 0.24 ^ab^	−74.98
GLB+VGN-10	3.4 ± 0.16 ^a^	4.1 ± 0.27 ^ab^	−20.78	5.0 ± 0.24 ^ab^	−49.11	5.7 ± 0.22 ^ab^	−70.08
GLB+VGN-20	3.6 ± 0.13 ^a^	4.7 ± 0.15 ^abc^	−31.29	5.8 ± 0.17 ^abc^	−62.28	7.1 ± 0.61 ^bc^	−97.80

Values are expressed as mean ± SEM of six animals in each group. ^a,b,c^ refer to significant changes at the corresponding time interval (*p* ≤ 0.05) with respect to NC, DC and GLB groups, respectively.

**Table 3:** Effect of GLB, VGN and their combinations on Hb and HbA1c levels in blood of STZ-induced rats.

Groups	Total Hemoglobin (g/dL)	HbA1c (%)
NC	14.6 ± 0.71	4.9 ± 0.34
DC	9.9 ± 0.25 ^a^	9.0 ± 0.32 ^a^
GLB	11.8 ± 0.37 ^ab^	6.7 ± 0.30 ^ab^
VGN-10	11.3 ± 0.32 ^ab^	7.1 ± 0.10 ^ab^
VGN-20	12.6 ± 0.24 ^ab^	5.9 ± 0.21 ^ab^
GLB+VGN-10	12.1 ± 0.44 ^ab^	6.3 ± 0.20 ^ab^
GLB+VGN-20	13.5 ± 0.39 ^bc^	5.2 ± 0.27 ^bc^

Values are expressed as mean ± SEM of six animals in each group. ^a,b,c^ refer to significant changes at *p* ≤ 0.05 with respect to NC, DC and GLB groups, respectively.

**Table 4:** Effect of GLB, VGN and their combinations on TG, TC, HDL-C and LDL-C levels in blood of STZ-induced rats.

Groups	TG (mg/dL)	TC (mg/dL)	HDL-C (mg/dL)	LDL-C (mg/dL)
NC	30.3 ± 1.14	43.3 ± 1.01	27.8 ± 1.05	14.6 ± 0.94
DC	56.3 ± 2.75 ^a^	72.0 ± 1.18 ^a^	16.6 ± 0.24 ^a^	36.9 ± 1.17 ^a^
GLB	41.4 ± 2.75 ^ab^	61.1 ± 1.71 ^ab^	19.5 ± 1.08 ^ab^	27.8 ± 1.21 ^ab^
VGN-10	45.3 ± 1.18 ^ab^	64.5 ± 2.61 ^ab^	18.3 ± 0.71 ^ab^	29.9 ± 0.66 ^ab^
VGN-20	34.8 ± 1.21 ^ab^	53.8 ± 2.99 ^ab^	22.6 ± 1.27 ^ab^	23.9 ± 1.51 ^ab^
GLB+VGN-10	37.9 ± 0.94 ^ab^	57.6 ± 2.82 ^ab^	20.4 ± 1.45 ^ab^	26.8 ± 1.28 ^ab^
GLB+VGN-20	27.9 ± 1.63 ^bc^	48.3 ± 2.32 ^bc^	25.6 ± 1.91 ^bc^	18.4 ± 1.65 ^bc^

Values are expressed as mean ± SEM of six animals in each group. ^a,b,c^ refer to significant changes at *p* ≤ 0.05 with respect to NC, DC and GLB groups, respectively.

**Table 5:** Effect of GLB, VGN and their combinations on SOD, GPx, CAT and MDA levels in pancreatic tissues of STZ-induced rats.

Groups	SOD (U/mg Protein)	GPx (U/mg Protein)	CAT (U/mg Protein)	MDA (nmol/g Tissue)
NC	51.7 ± 2.43	8.7 ± 0.42	14.3 ± 0.98	30.2 ± 1.86
DC	26.7 ± 0.79 ^a^	4.8 ± 0.22 ^a^	5.8 ± 0.12 ^a^	54.9 ± 1.14 ^a^
GLB	31.4 ± 1.93 ^ab^	5.7 ± 0.33 ^ab^	7.2 ± 0.58 ^ab^	48.2 ± 2.76 ^ab^
VGN-10	34.1 ± 2.14 ^ab^	6.3 ± 0.39 ^ab^	7.8 ± 0.39 ^ab^	45.4 ± 1.41 ^ab^
VGN-20	39.7 ± 3.68 ^ab^	7.1 ± 0.55 ^ab^	9.1 ± 0.73 ^ab^	40.8 ± 2.89 ^ab^
GLB+VGN-10	37.8 ± 2.26 ^ab^	6.5 ± 0.20 ^ab^	8.9 ± 0.68 ^ab^	43.6 ± 2.53 ^ab^
GLB+VGN-20	46.7 ± 2.85 ^bc^	7.6 ± 0.30 ^bc^	12.1 ± 0.31 ^bc^	36.4 ± 2.62 ^bc^

Values are expressed as mean ± SEM of six animals in each group. ^a,b,c^ refer to significant changes at *p* ≤ 0.05 with respect to NC, DC and GLB groups, respectively.

## Data Availability

Not applicable.

## Supplementary Materials

The following supporting information can be downloaded at: https://www.mdpi.com/article/10.3390/ijms232415856/s1.
