# Supplementary material for: Vulgarin, a Sesquiterpene Lactone from Artemisia judaica, Improves the Antidiabetic Effectiveness of Glibenclamide in Streptozotocin-Induced Diabetic Rats via Modulation of PEPCK and G6Pase Genes Expression
Source: Int J Mol Sci. 2022 Dec 13;23(24):15856. doi: 10.3390/ijms232415856 (PMC9781739; doi:10.3390/ijms232415856)
Supplement: Supplementary file 1 [file ijms-23-15856-s001.zip › ijms-2066788-supplementary.pdf]

Vulgarin, a sesquiterpene lactone from *Artemisia judaica* improves the antidiabetic effectiveness of glibenclamide in streptozotocin-induced diabetic rats via modulation of PEPCK and G6Pase genes expression

Hassan N. Althurwi [a], Gamal A. Soliman [a,b], Rehab F. Abdel-Rahman [c], Reham M. Abd-Elsalam[d], Hanan A. Ogaly [e,f], Mohammed H. Alqarni [g], Maged S. Abdel-Kader [g,h,*]

[a] *Department of Pharmacology, College of Pharmacy, Prince Sattam Bin Abdulaziz University, Al-Kharj 11942, Saudi Arabia*
[b] *Department of Pharmacology, College of Veterinary Medicine, Cairo University, Egypt*
[c] *Department of Pharmacology, National Research Centre, Egypt*
[d] *Department of Pathology, College of Veterinary Medicine, Cairo University, Egypt*
[e] *Department of Chemistry, College of Science, King Khalid University, Abha, Saudi Arabia*
[f] *Department of Biochemistry, College of Veterinary Medicine, Cairo University, Egypt*
[g] *Department of Pharmacognosy, College of Pharmacy, Prince Sattam Bin Abdulaziz University, Al-Kharj 11942, Saudi Arabia*
[h] *Department of Pharmacognosy, College of Pharmacy, Alexandria University, Alexandria 21215, Egypt*

Vulgarin (VGN): Colourless crystals, m.p. 175 ^{0}C; $[\alpha]^{25}_D$ = + 43; UV λmax 216 nm; ^{1}H and ^{13}C NMR Table S1; HRESIMS $[M+1]^+$ *m/z* 265.1429 (calcd for $C_{15}H_{20}O_4$+H, 265.1440, 6%), $[M+Na]^+$ *m/z* 287.1247 (calcd for $C_{15}H_{20}O_4$+Na, 287.1259, 100%), $[M-1]^+$ *m/z* 263.1287 (calcd for $C_{15}H_{20}O_4$-H, 263.1283, 100%).

4-Epivulgarin (VGN): Colourless crystals, m.p. 193 ^{0}C; $[\alpha]^{25}_D$ = + 82; UV λmax 215 nm; ^{1}H and ^{13}C NMR Table S1; HRESIMS $[M+1]^+$ *m/z* 265.1429 (calcd for $C_{15}H_{20}O_4$+H, 265.1440, 55%), $[M+Na]^+$ *m/z* 287.1248 (calcd for $C_{15}H_{20}O_4$+Na, 287.1259, 100%), $[M-1]^+$ *m/z* 263.1288 (calcd for $C_{15}H_{20}O_4$-H, 263.1288, 100%).

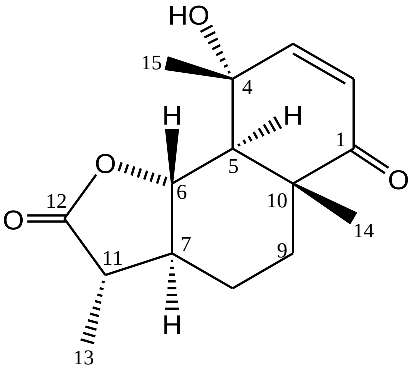

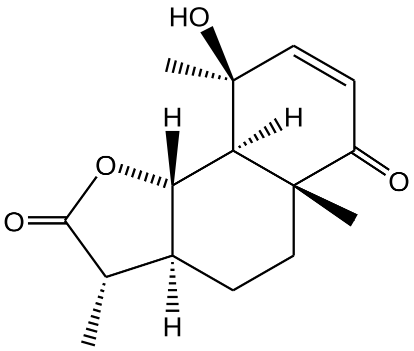

Table S1. ^{1}H and ^{13}C NMR data for VGN and eVGN ($CDCl_3$, δ in ppm).

	**Vulgarin (VGN)**		**4-Epivulgarin (eVGN)**	
	**^{1}H**	**^{13}C**	**^{1}H**	**^{13}C**
**1**	**-**	201.80	**-**	203.64
**2**	5.72 (d, *J*= 10.3 Hz)	125.31	5.72 (d, *J*= 10.3 Hz)	124.64
**3**	6.47 (d, *J*= 10.3 Hz)	152.03	6.42 (d, *J*= 10.3 Hz)	151.22
**4**	**-**	69.91	**-**	67.76
**5**	2.27 (d, *J*= 10.95 Hz)	54.39	1.88 (d, *J*= 10.95 Hz)	50.97
**6**	4.06 (t, *J*= 11 Hz)	79.41	4.25 (t, *J*= 10.75 Hz)	79.34
**7**	1.54 (m)	52.19	1.55 (m)	52.20
**8**	1.37 (m), 1.85 (m)	22.53	1.39 (m), 1.81 (m)	22.61
**9**	1.37 (m), 1.85 (m)	34.01	1.39 (m), 1.81 (m)	32.52
**10**	**-**	46.25	**-**	45.97
**11**	2.22 (m)	40.39	2.21 (h, *J*= 6.85 Hz)	40.71
**12**	**-**	178.43	**-**	179.46
**13**	1.09 (d, *J*= 6.95 Hz)	12.39	1.09 (d, *J*= 6.95 Hz)	12.39
**14**	1.07 (s)	19.71	1.20 (s)	20.65
**15**	1.41 (s)	23.56	1.44 (s)	31.09
**OH**	2.95 (s)	-	3.03 (s)	-

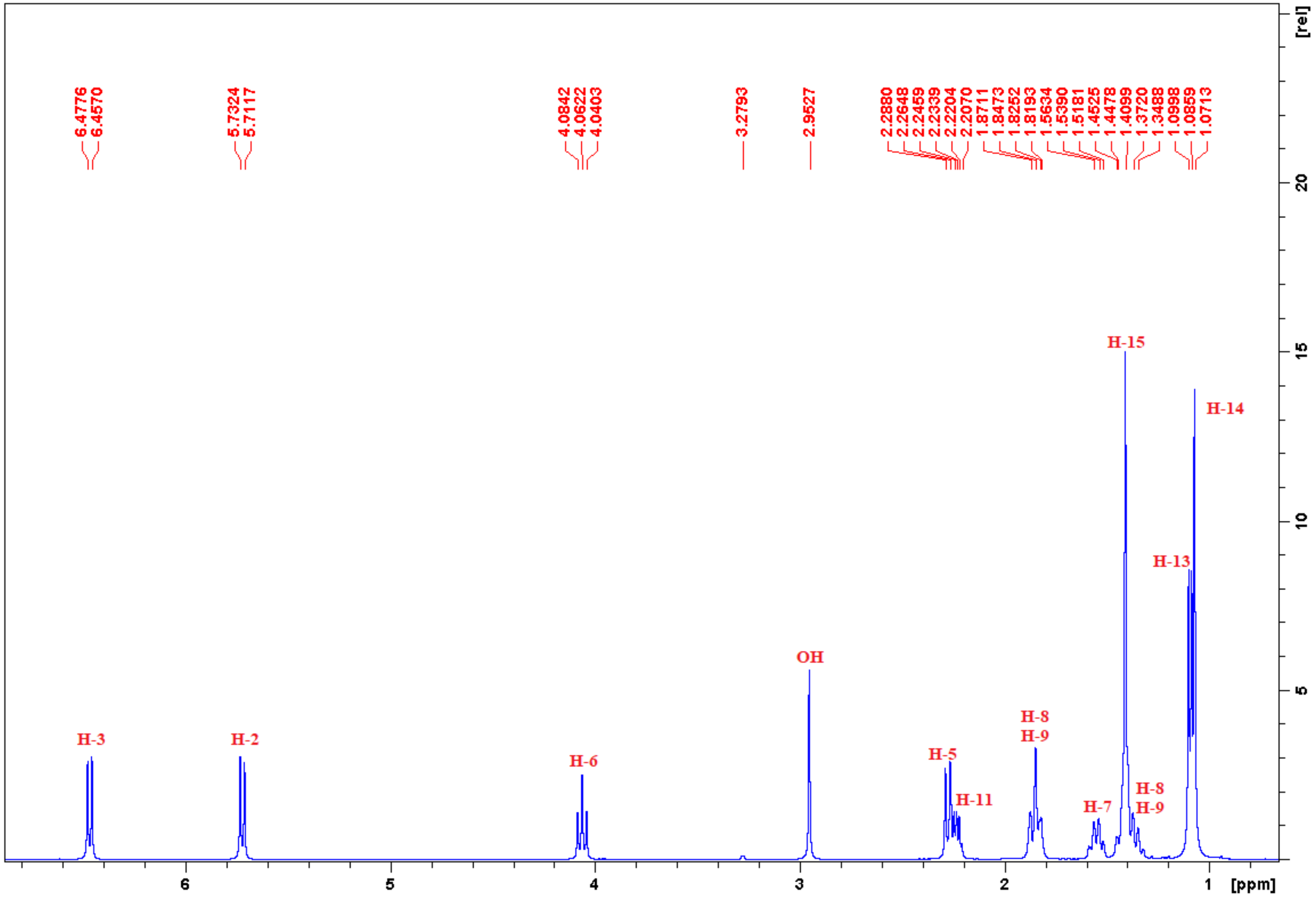

Figure S1: ^{1}H NMR data for VGN.

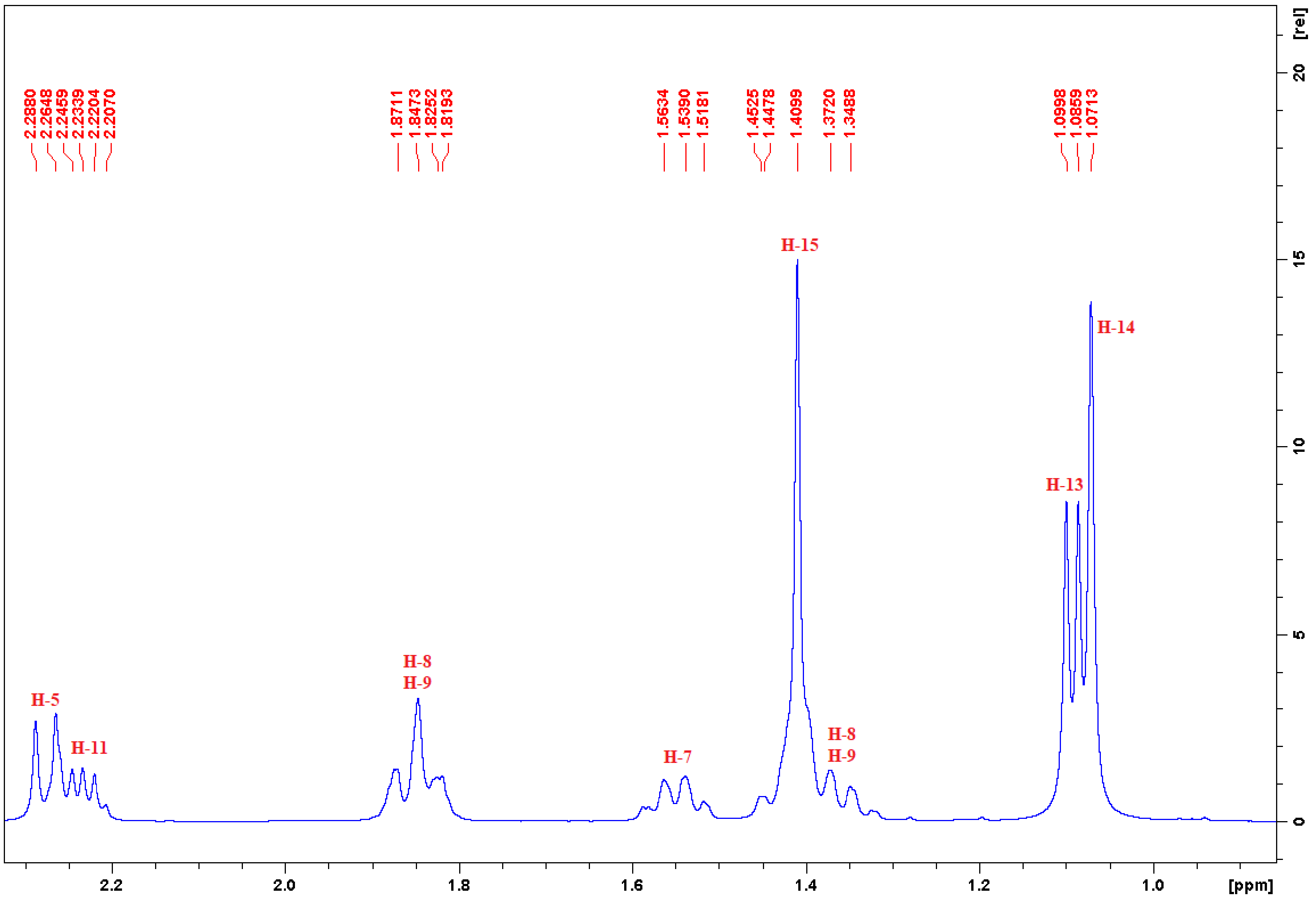

Figure S2: ^{1}H NMR data for VGN (Exp.).

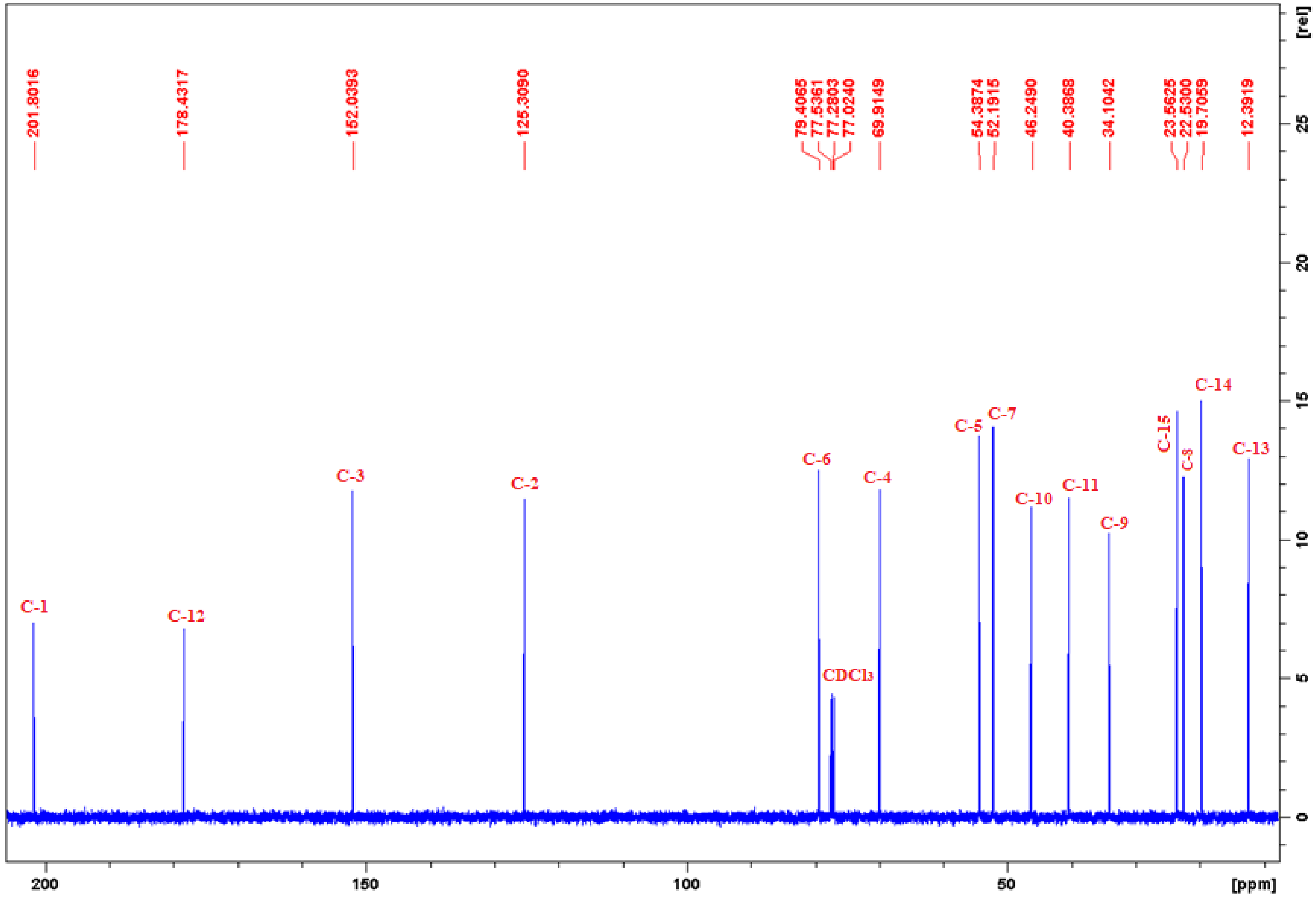

Figure S3: ^{13}C NMR data for VGN.

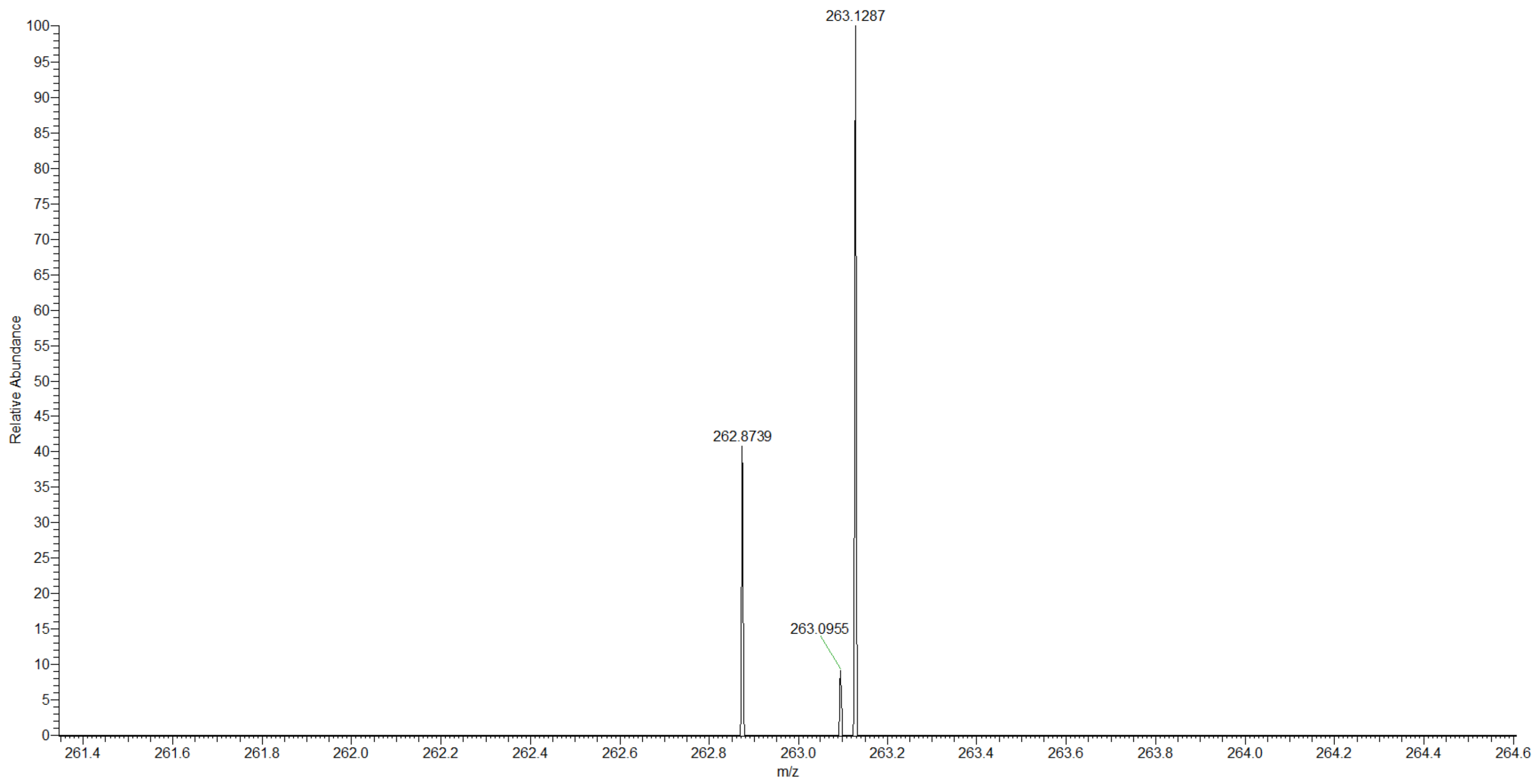

Figure S4: HRESIMS data of VGN (negative mode).

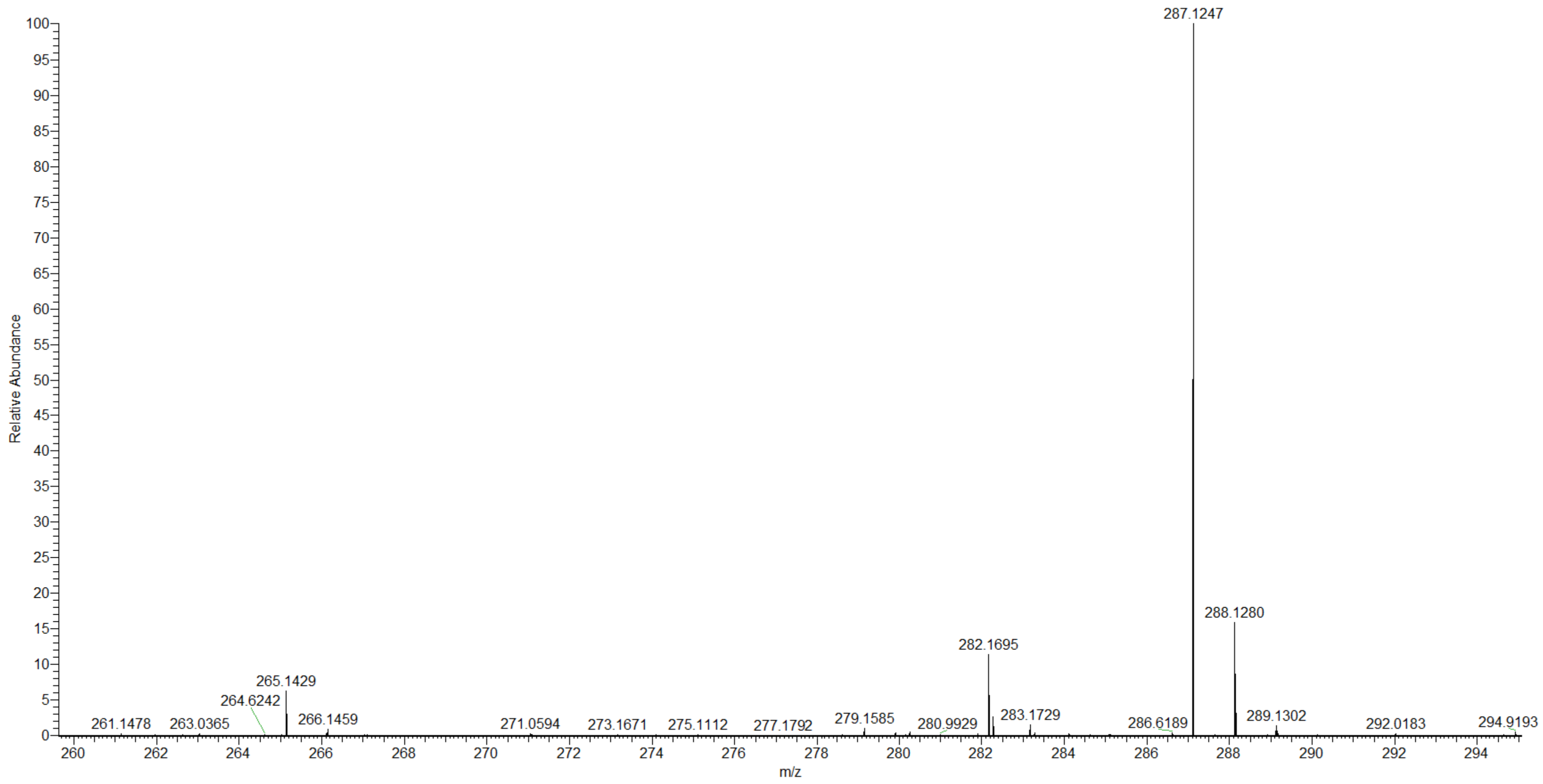

Figure S5: HRESIMS data of VGN (positive mode).

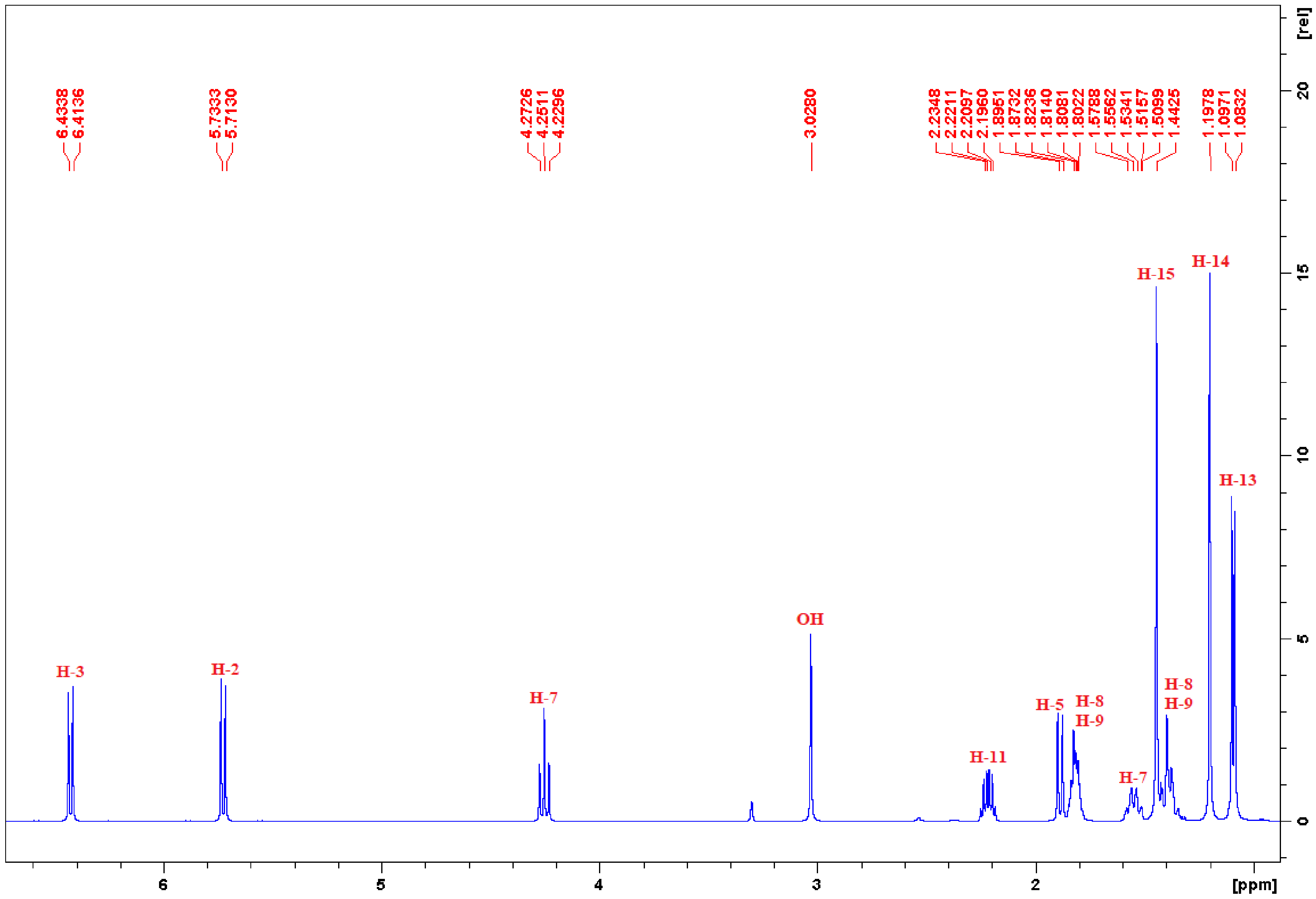

Figure S6: ^{1}H NMR data for eVGN.

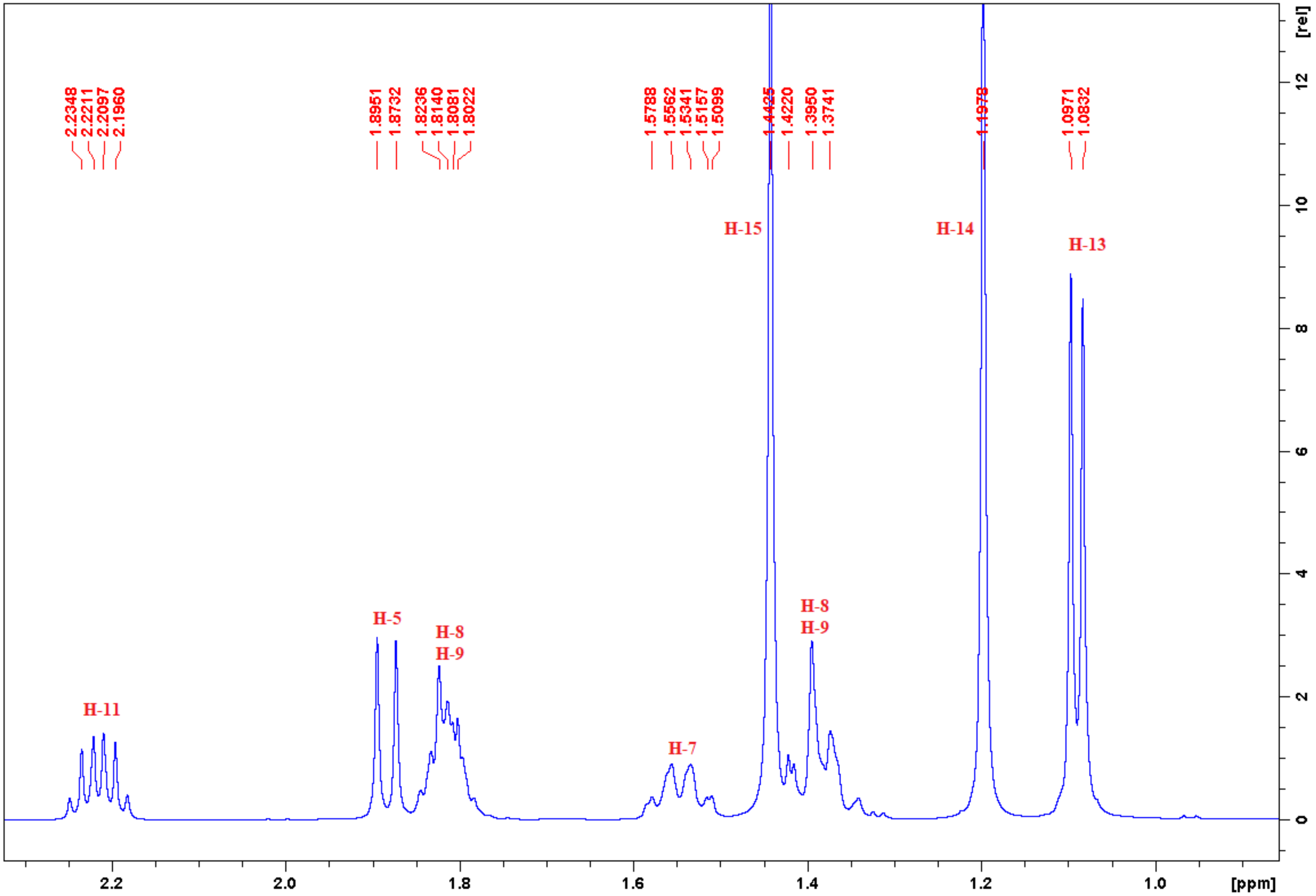

Figure S7: ^{1}H NMR data for eVGN (Exp.).

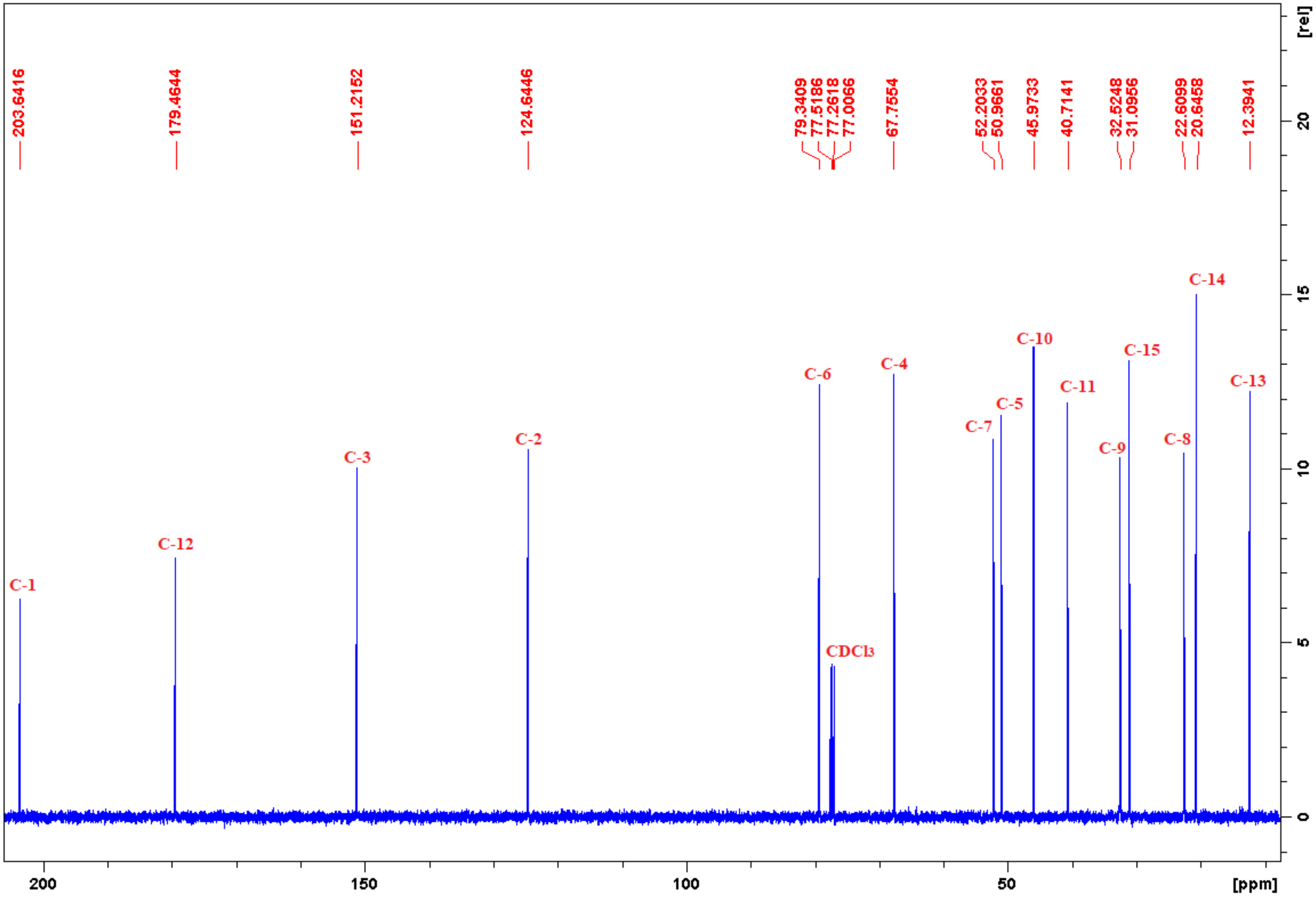

Figure S8: ^{13}C NMR data for eVGN.

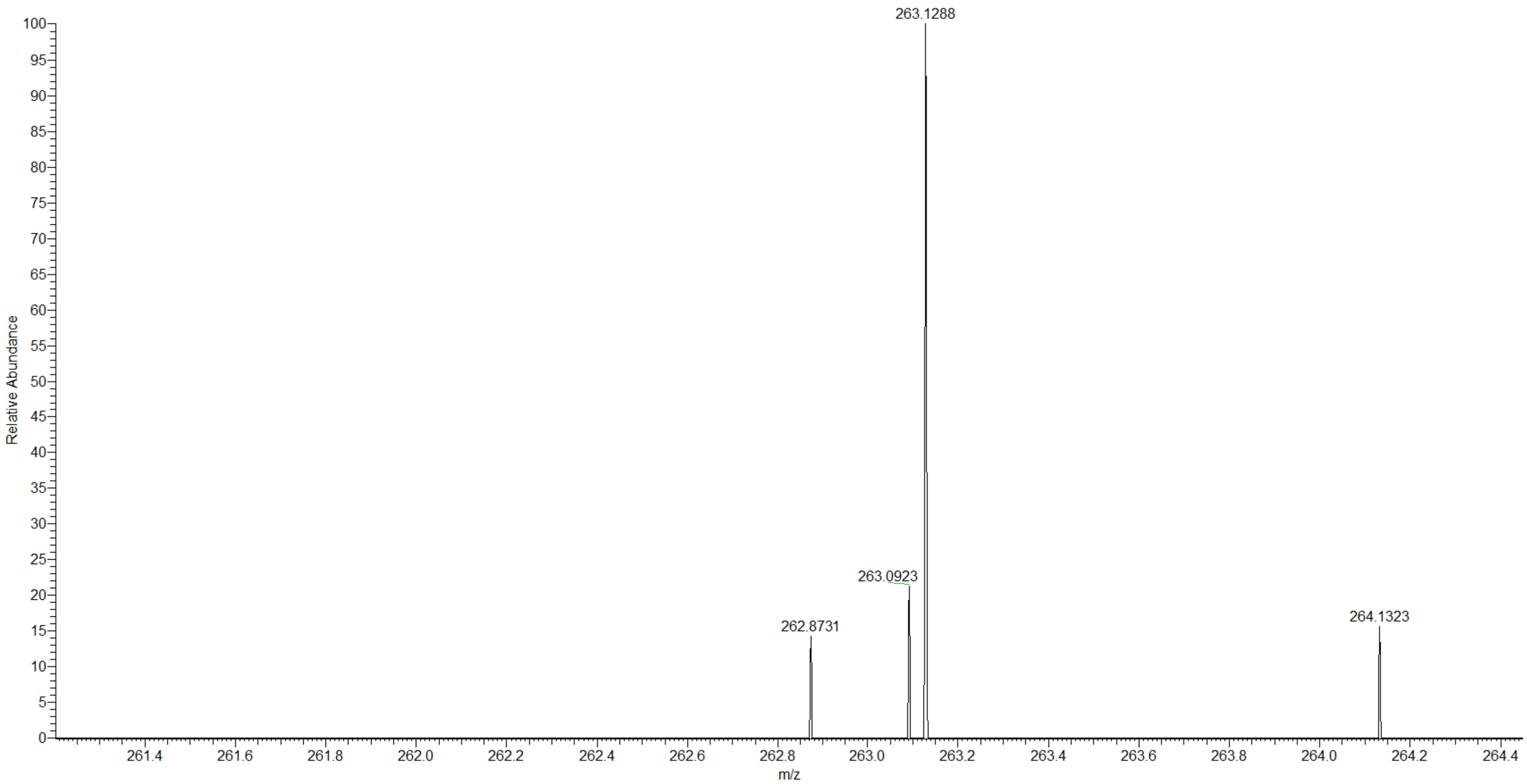

Figure S9: HRESIMS data of eVGN (negative mode).

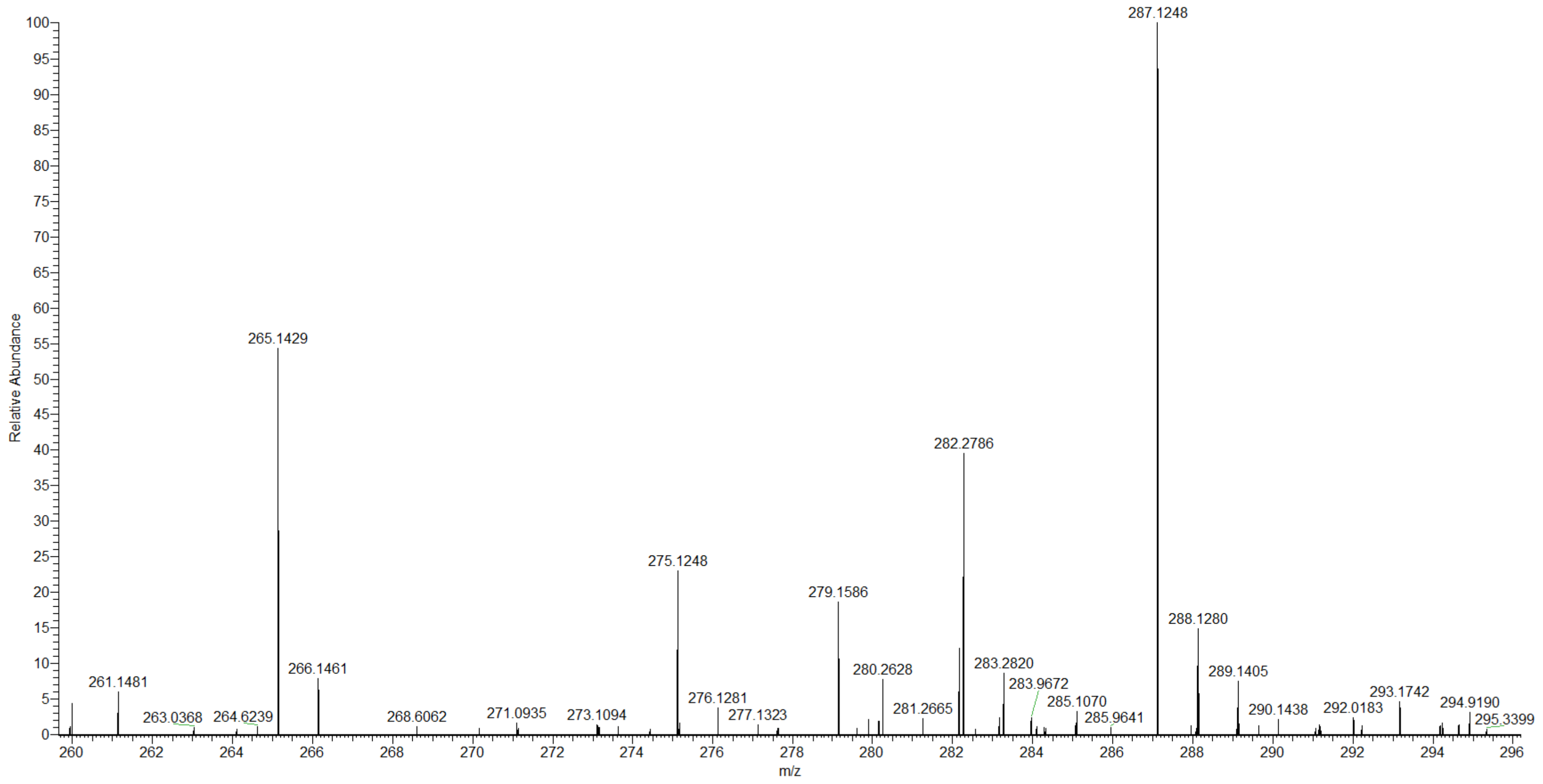

Figure S10: HRESIMS data of eVGN (positive mode).
